# A Dopamine Detection Sensor Based on Au-Decorated NiS_2_ and Its Medical Application

**DOI:** 10.3390/molecules29122925

**Published:** 2024-06-20

**Authors:** Chongchong Ma, Yixuan Wen, Yuqing Qiao, Kevin Z. Shen, Hongwen Yuan

**Affiliations:** 1 State Key Laboratory of Metastable Materials Science and Technology, Yanshan University, Qinhuangdao 066004, China; macc321@163.com (C.M.); yixuanw001@163.com (Y.W.); qiaoyq@ysu.edu.cn (Y.Q.); 2 Department of Biology Texas, A&M University, College Station, TX 77483, USA; kevinxshen@gmail.com; 3 School of Traditional Chinese Medicine, Capital Medical University, Beijing 100069, China

**Keywords:** dopamine sensor, NiS_2_, Au decorated, hydrothermal

## Abstract

This article reports a simple hydrothermal method for synthesizing nickel disulfide (NiS_2_) on the surface of fluorine-doped tin oxide (FTO) glass, followed by the deposition of 5 nm Au nanoparticles on the electrode surface by physical vapor deposition. This process ensures the uniform distribution of Au nanoparticles on the NiS_2_ surface to enhance its conductivity. Finally, an Au@NiS_2_-FTO electrochemical biosensor is obtained for the detection of dopamine (DA). The composite material is characterized using transmission electron microscopy (TEM), UV-Vis spectroscopy, X-ray diffraction, and X-ray photoelectron spectroscopy. The electrochemical properties of the sensor are investigated using cyclic voltammetry (CV), differential pulse voltammetry (DPV), and time current curves in a 0.1 M PBS solution (pH = 7.3). In the detection of DA, Au@NiS_2_-FTO exhibits a wide linear detection range (0.1~1000 μM), low detection limit (1 nM), and fast response time (0.1 s). After the addition of interfering substances, such as glucose, L-ascorbic acid, uric acid, CaCl_2_, NaCl, and KCl, the electrode potential remains relatively unchanged, demonstrating its strong anti-interference capability. It also demonstrates strong sensitivity and reproducibility. The obtained Au@NiS_2_-FTO provides a simple and easy-to-operate example for constructing nanometer catalysts with enzyme-like properties. These results provide a promising method utilizing Au coating to enhance the conductivity of transition metal sulfides.

## 1. Introduction

DA is a monoamine neurotransmitter found in the human brain and kidneys. It plays an important role in brain control, the immune system, and the renal system. It is crucial for human learning, cognition, motor behavior, and hormone production [1,2,3,4]. The DA level adjustment can lead to various neurological disorders, such as schizophrenia, Parkinson’s disease, epilepsy, increased heart rate, arrhythmia, elevated blood pressure, and abnormalities in the cardiovascular system [5,6,7,8]. Therefore, determining and monitoring DA levels in real time is quite significant and can be used not only for early diagnosis of diseases but also for evaluating the effectiveness of various drugs in DA manipulation. Moreover, it helps to deepen our understanding of the mechanisms of DA function and better treatment of various diseases [9].

Traditional methods for detecting DA include blood tests and 24 h urine sampling. Usually, these methods require specialized technical training and laboratory equipment, including high-performance liquid chromatography, fluorescence spectroscopy, enzyme analysis, mass spectrometry, and capillary electrophoresis, which are greatly limited due to the high cost and operationally complex [10,11,12,13,14]. Recently, electrochemical sensing methods developed fast, offering advantages such as simplicity, low cost, ease of use, rapid analysis, and high sensitivity to compensate for the limitations of traditional methods. Currently, these sensors have been widely used in various fields such as biological analysis, clinical diagnosis, drug screening, and environmental monitoring. For instance, they can be utilized to detect biomolecules, like blood glucose, cholesterol, and tumor markers, enabling diabetes management, cardiovascular disease diagnosis, and early cancer detection. Moreover, electrochemical biosensors can also monitor heavy metal ions, organic pollutants, and microorganisms in the environment, contributing to environmental protection and food safety control. Ferlazzo et al. developed a yttria-stabilized zirconia electrochemical sensor for the precise determination of tyrosine [15]; Ahmed et al. prepared In_2_O_3_·ZnO@MC for dopamine detection with a detection limit of 0.024 μM [16]. These sensors have been widely applied in various electrocatalytic fields, such as supercapacitors and lithium-ion batteries [17,18]. Moreover, they can be integrated into miniaturized devices for use in medical experiments and procedures [19,20,21]. Thus, plenty of electrode materials are developed for the detection of DA. Electrode materials must be able to detect and differentiate other molecules, providing a distinct peak for DA. Meanwhile, they also need high anti-interference properties, enabling independent detection of DA amidst the presence of multiple substances without interference. Additionally, they should have a wide detection range to meet the medical research needs regarding DA levels [22,23,24,25,26].

Currently, transition metal dichalcogenides (TMDs) are receiving significant attention due to their distinctive structural features, morphologies, conductivity properties, and their role in facilitating electron transfer as narrow bandgap semiconductors [27,28,29]. Among the amount of TMDs, nickel disulfide (NiS_2_) attracts much attention due to its high electrochemical activity and relatively low synthesis costs. NiS_2_ is a good electrocatalytic material, but its poor conductivity largely limits its application [30]. Its intrinsic conductivity is modest, posing limitations on electron transfer within electrochemical biosensor systems [31,32]. Therefore, enhancing electrical conductivity is crucial for improving catalytic activity. Referring to previous catalytic materials, Au coating is a very effective technique. Kim et al. developed a method of Au nanoparticle modification of MoS_2_, significantly improving the material’s charge transfer [33]. Atta et al. doped Au into polyvinyl alcohol/carboxymethylcellulose, greatly enhancing the material’s electrical conductivity [34]. However, this study presents a novel approach wherein nickel disulfide is directly synthesized via a one-step hydrothermal method. We coated Au on the surface of NiS_2_ using physical vapor deposition in this study, significantly enhancing its electrical conductivity. A 5 nm Au film is deposited onto the surface using a physical vapor deposition (PVD) system, resulting in the formation of Au@NiS_2_. Furthermore, through tests, such as CV, it was found that the electrocatalytic biosensing performance is greatly enhanced. The surface-bound Au particles serve to augment the conductivity of NiS_2_. This composite system not only exhibits enhanced stability but also facilitates electron transfer subsequent to DA molecule signal recognition. Thus, the Au@NiS_2_ composite material offers considerable potential for advancing the development of high-sensitivity electrochemical biosensors, representing a promising candidate for the next generation of materials in this field.

## 2. Results and Discussion

### 2.1. Electrochemical Characterization

**Figure 1:**
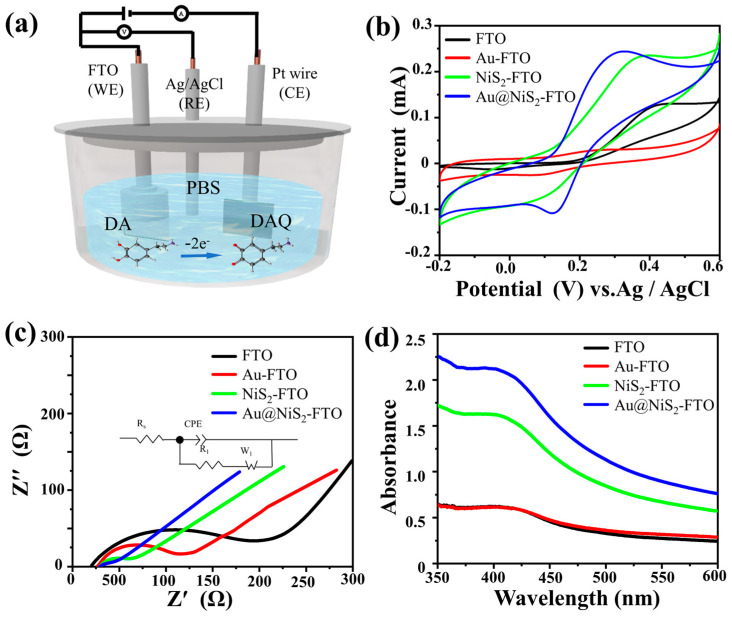
(**a**) Schematic of the electrochemical testing setup; (**b**) CVs of bare FTO, Au-FTO, NiS_2_-FTO, and Au@NiS_2_-FTO in 1 mM DA in 0.01 M PBS (pH = 7.3) at a scan rate of 0.1 V s^−1^; (**c**) Nyquist plots of bare FTO, Au-FTO, FTO-NiS_2_, and FTO-Au@NiS_2_ in 0.1 M KCl containing 0.5 mM Fe(CN${)}_{6}^{3-}$ and Fe(CN${)}_{6}^{4-}$; (**d**) the UV–Vis absorption spectra of 1 mM DA in 0.1 M PBS (pH = 7.3) before and after applying a voltage of 0.3 V.

In the experiment, we first compare the electrochemical behavior of Au@NiS_2_-FTO, NiS_2_-FTO, Au-FTO, and pure FTO, respectively. It has already been reported that the oxidation reduction reaction of DA involves a two-electron transfer process, converting DA to DA quinone [35,36]. The schematic of the electrochemical setup, as depicted in Figure 1a, illustrates the foundation for exploring the process of the electrochemical reaction process. This experiment was conducted employing a sophisticated three-electrode system integrated into the CHI660E electrochemical workstation. In this study, an FTO substrate (10 × 6 mm) was selected as the pivotal working electrode (WE). Complementing this, a platinum plate was designated as the auxiliary electrode (CE), while an Ag/AgCl electrode played the indispensable reference electrode (RE). Initially, DA was meticulously dissolved in 0.1 M PBS (pH = 7.3) to establish a standardized 1 mM DA solution. Subsequently, the distinct performances of the various electrodes were examined through the application of cyclic voltammetry. By scrutinizing the cyclic voltammetry outcomes, it became feasible to gauge the intrinsic electrocatalytic prowess of the DA sensing electrode, primarily by assessing the DA oxidation peak potential. This pivotal metric served as a cornerstone for evaluating the efficacy and sensitivity of the electrode in facilitating the electrochemical detection of DA.

As shown in Figure 1b, the pristine NiS_2_-FTO exhibits weak catalytic ability for the detection of DA, with a low peak and almost no reaction with DA observed for the original FTO and Au-FTO. In contrast, Au@NiS_2_-FTO shows a significant current response to DA, with an oxidation potential reaching Epa = 0.3 V. The Au nanoparticles effectively enhance the electrocatalytic performance of NiS_2_.

It is found that the Au coating significantly enhances the activity of NiS_2_, and the large number of exposed active sites facilitates interaction with DA, thereby improving electrochemical performance [37,38]. As expected, the oxidation peak current of Au@NiS_2_-FTO is 1.5 times higher than that of NiS_2_-FTO, indicating that Au@NiS_2_-FTO exhibits stronger electrocatalytic activity for DA detection. Compared to NiS_2_-FTO, Au-FTO, and FTO, the peak separation (ΔEp) of Au@NiS_2_-FTO is significantly reduced, suggesting improved reaction kinetics. Additionally, using a solution containing 5 mM K_3_[Fe(CN)_6_], 5 mM K_4_[Fe(CN)_6_], and 0.1 M KCl as the redox probe solution, the interface performance of different sensors was tested using electrochemical impedance spectroscopy (EIS). By fitting the EIS spectra with equivalent circuits (Figure 1c), the test data are shown in Table 1. The charge transfer resistance of the Au@NiS_2_-FTO electrode is significantly lower than that of NiS_2_-FTO, Au-FTO, and FTO, with the sequence being FTO > Au-FTO > NiS_2_-FTO. This is consistent with the CV results, indicating that Au@NiS_2_-FTO has a better electron transfer rate and is, therefore, more suitable for DA detection. To further verify the oxidation reduction reaction between DA and DA quinone, we conducted tests using UV–visible absorption spectroscopy. In Figure 1b, it is observed that the oxidation peak of DA is at 0.3 V. Therefore, in this study, chronoamperometry was used to electrolyze 1 mM DA by different electrodes in three-electrode systems for 10 min, followed by testing the collected reaction solution, as shown in Figure 1d.

In Figure 1d, it can be observed that FTO and Au-FTO did not exhibit significant absorption peaks. However, after electrolyzing for 10 min, a peak corresponding to the characteristic absorption peak of DA quinone was observed at around 400 nm for NiS_2_-FTO and Au@NiS_2_-FTO. Moreover, the absorption peak intensity of Au@NiS_2_-FTO was significantly higher than that of NiS_2_-FTO, indicating that the catalytic activity of Au@NiS_2_-FTO is much higher than that of NiS_2_-FTO. Additionally, no characteristic absorption peak of DA quinone was observed in the spectrum after electrolyzing for 10 min using Au-FTO, indicating that only Au@NiS_2_-FTO exhibits catalytic activity.

### 2.2. Structure and Morphology Characterization

**Figure 2:**
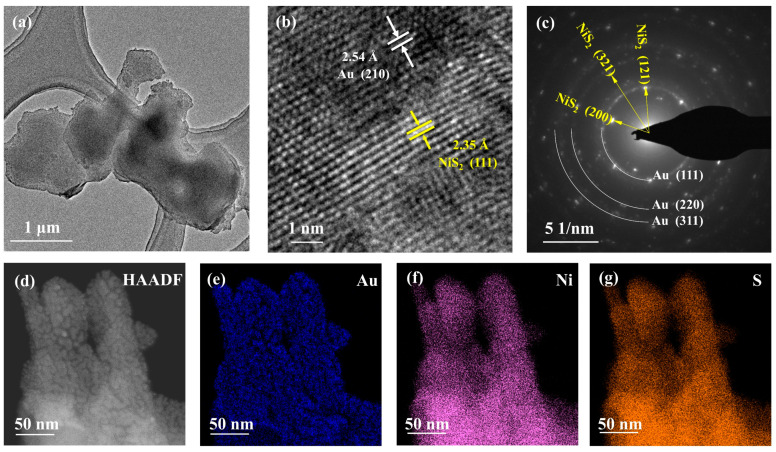
(**a**) TEM images of Au@NiS_2_; (**b**) HRTEM images of Au@NiS_2_; (**c**) SAED pattern of Au@NiS_2_; (**d**) scanning HAADF-STEM image of Au@NiS_2_ and corresponding elemental mapping of (**e**) Au, (**f**) Ni, and (**g**) S.

The electrocatalytic performance of materials is intricately intertwined with their underlying structure and morphology. In our experiment, we scrutinized the morphology and structure of the Au@NiS_2_ material through transmission electron microscopy (TEM) and High-Resolution Transmission Electron Microscopy (HRTEM). Figure 2b vividly illustrates the characteristics of NiS_2_, showcasing a remarkable two-dimensional nanosheet-like structure distribution, with Au nanofilm equably dispersed across the surface of NiS_2_. Upon examination through HRTEM, the lattice spacing of 2.35 Å was linked to the (111) crystal plane of NiS_2_, while the lattice spacing of 2.54 Å corresponded to the (210) crystal plane of Au. This inspection unequivocally demonstrated the seamless integration of Au with NiS_2_, a pivotal aspect of catalytic efficacy. Further insights were gleaned from the Selected Area Electron Diffraction (SAED) pattern, revealing distinctive rings corresponding to the face-centered cubic structure reflections of Au, notably the (111), (220), and (311) planes, alongside the crystal planes of NiS_2_, such as (121), (220), and (311). Additionally, the Energy-Dispersive X-ray Spectroscopy (EDX) spectra showcased the elemental distribution, and Au prominently adhered to the surface of NiS_2_, showcasing a robust and well-integrated bond between the two materials. This comprehensive characterization not only elucidated the structure but also underscored the synergistic relationship between Au and NiS_2_ in the context of electrocatalytic applications.

**Figure 3:**
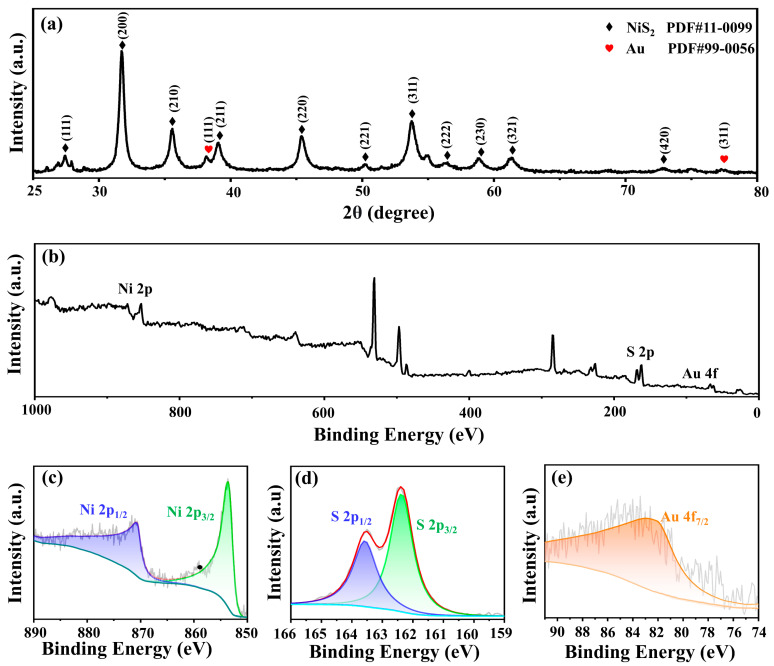
(**a**) XRD patterns of Au@NiS_2_. (**b**) XPS survey spectra of Au@NiS_2_ and the high-resolution spectra of Au@NiS_2_ in the energy range of (**c**) Ni 2p, (**d**) S 2p, and (**e**) Au 4f.

Figure 3a shows the X-ray diffraction (XRD) analysis of Au@NiS_2_ into the structure of the composite material. Notably, the peaks corresponding to the presence of Au nanoparticles are discernible at 38.18° and 77.56°. By referencing the standard PDF card (PDF#99-0056), these peaks align precisely with the (111) and (311) crystal planes of Au, elucidating the crystalline orientation of the Au nanoparticles within the composite. Simultaneously, the XRD pattern also reveals characteristic peaks associated with NiS_2_ at 27.25°, 31.59°, and 35.31°. These peaks are in direct correspondence with the (111), (200), and (210) crystal planes of NiS_2_, as validated by the reference standard PDF card (PDF#11-0099).

The comprehensive characterization of the Au@NiS_2_ composite material extended to the elemental composition and electronic states through X-ray photoelectron spectroscopy (XPS) analysis. Figure 3b shows the XPS spectrum of Au@NiS_2_, unveiling distinctive peaks corresponding to the elemental constituents of Au, Ni, and S. Within the Au 4f spectrum, a peak emerges at 83.4 eV, indicative of the electronic configuration of Au within the composite. Transitioning to the Ni 2p spectrum, the presence of two distinct spin–orbit peaks, 2p_3/2_ and 2p_1/2_, is prominently observed at 855 and 872 eV. Furthermore, the corresponding peaks for these spin–orbit orbitals manifest at 162.5 and 163.8 eV, providing evidence that nickel predominantly exists in the Ni^2+^ oxidation state. This finding aligns with the structural revelations from the XRD analysis, thereby establishing a coherent link between the crystallographic characteristics and the electronic states of Ni within the Au@NiS_2_ composite material [39].

### 2.3. Sensor Application

**Figure 4:**
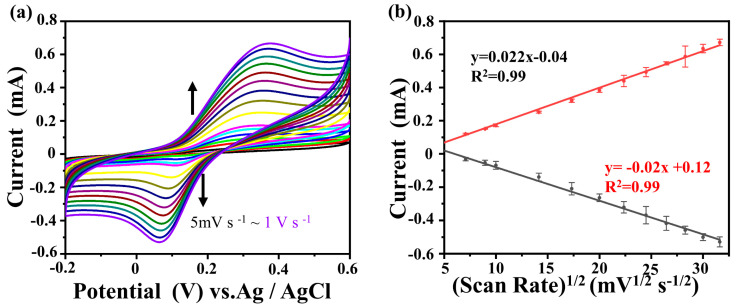
(**a**) CV curves of the Au@NiS_2_-FTO-modified electrode in 0.1 M PBS (pH = 7.3) containing 1 mM DA by varying the scan rates from 5 mV s^−1^ to 1 V s^−1^; (**b**) Ipa and Ipc vs. v^1/2^**.**

To delve deeper into the intricate dynamics of the electrochemical reaction kinetics between DA and Au@NiS_2_-FTO, as depicted in Figure 4a, a meticulous exploration was undertaken, employing a three-electrode system immersed in a 0.1 M PBS (pH = 7.3) solution as the electrolyte medium. The experimental investigation entailed the application of cyclic voltammetry, encompassing a scan rate range spanning from 5 mV s^−1^ to 1 V s^−1^ and a voltage window extending from −0.2 to 0.6 V. The outcomes of these rigorous cyclic voltammetry experiments unveiled that the oxidation reduction peaks of DA exhibited a notable augmentation concomitant with the escalation of the scan rate. Simultaneously, a discernible shift in the oxidation potential of DA was observed, indicating a quasi-reversible nature of the electrochemical reaction within this system. These findings collectively underscore the dynamic interplay between DA and the Au@NiS_2_-FTO composite electrode, shedding light on the kinetic intricacies governing the electrochemical processes at play. The anodic peak current (Ipa) and cathodic peak current (Ipc) are linearly related to the square root of the scan rate, and the linear regression equations are as follows:

Ipa = 0.022x − 0.04 (R^2^ = 0.99), (1)

Ipc = −0.02x + 0.12 (R^2^ = 0.99), (2)

The slope of this regression curve indicates that this electrochemical behavior is mainly controlled by diffusion, and its reversibility is good [40,41,42]. In the oxidation reaction, DA is converted to DA quinone, which is then reduced back to DA in the electrochemical reaction [43].

**Figure 5:**
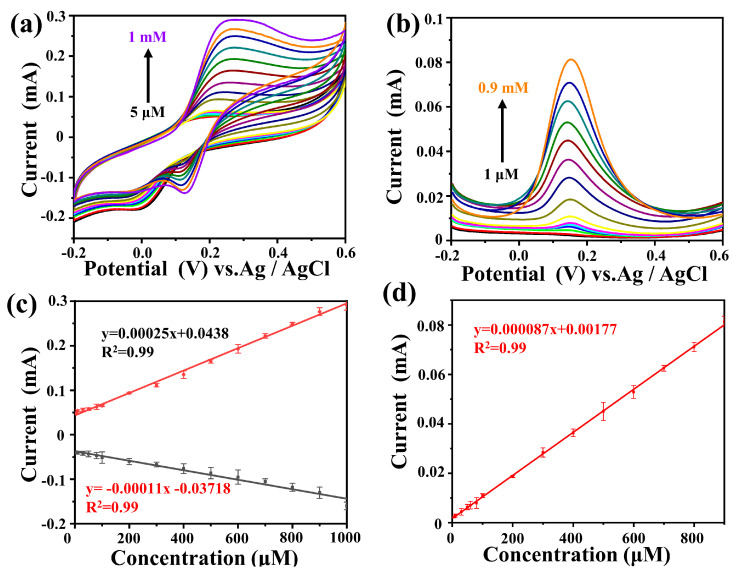
(**a**) CV curves of Au@NiS_2_-FTO in 0.1 M PBS (pH = 7.3) containing various concentrations of DA (5–1 mM) at a scan rate of 0.1 V s^−1^; (**b**) DPV curves of Au@NiS_2_-FTO in 0.1 M PBS (pH = 7.3) containing various concentrations of DA (1–0.9 mM); (**c**) linear relationship between peak potential and concentration in CV; (**d**) linear relationship between peak potential and concentration in DPV.

Subsequently, in order to further determine the response of Au@NiS_2_-FTO to different concentrations of DA solutions, a standard three-electrode system was used with 0.1 M PBS (pH = 7.3) as the electrolyte. Cyclic voltammetry was performed within the voltage range of −0.2 V to 0.6 V at the same scan rate (0.1 V s^−1^) to test the response signal to the addition of different concentrations of DA, as shown in Figure 5a. The linear regression equations obtained from cyclic voltammetry are as follows:

Ipa = 0.00025x + 0.0438 (R^2^ = 0.99), (3)

Ipc = −0.00011x − 0.03718 (R^2^ = 0.99), (4)

In (3) and (4), it can be seen that Au@NiS_2_-FTO exhibits a good response signal to DA, and under the stimulation of different concentrations of DA, the oxidation reduction peak remains unchanged, indicating that Au@NiS_2_-FTO owns a stable ability for the detection of DA. The DA concentration range satisfied by this regression curve is 5 μM to 1 mM, indicating that Au@NiS_2_-FTO can achieve broad-range DA detection.

Moreover, differential pulse voltammetry (DPV) is an electrochemical analysis technique commonly used to measure electrochemically active substances in solution. The testing method involves applying a series of pulse voltages on an electrochemical electrode and measuring the current response caused by these pulse voltages. DPV is a widely used electrochemical measurement method in sensor analysis, known for its high sensitivity and selectivity [44]. DPV was used to test its sensitivity by adding different concentrations of DA within the same voltage range. Similarly, in a three-electrode system with 0.1 M PBS (pH = 7.3) as the electrolyte, DPV was performed with a voltage window from −0.2 V to 0.6 V, a step potential of 4 mV, an amplitude of 50 mV, and a pulse period of 0.2 s. The obtained data are shown in Figure 5d, and the linear regression equation is as follows:

Ipc = −0.000087x + 0.00177 (R^2^ = 0.99), (5)

In Figure 5b, it can be seen that the linear detection range of DPV is from 1 μM to 0.9 mM.

**Figure 6:**
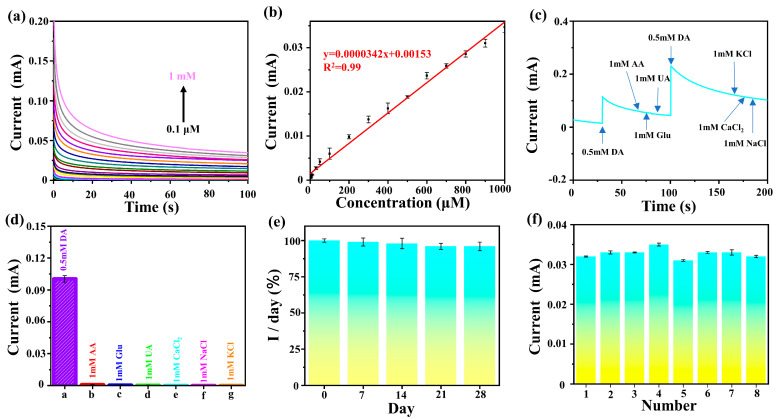
(**a**) The curves of Au@NiS_2_-FTO in 0.1 M PBS (pH = 7.3) containing various concentrations of DA (0.1–1 mM) at 0.3 V; (**b**) linear relationship between peak potential and concentration in CV; (**c**) amperometric response obtained at Au@NiS_2_-FTO for the successive addition of 0.5 mM DA, 1 mM glucose, 1 mM AA, 1 mM UA, and 0.5 mM DA into 0.1 mM PBS (pH = 7.3); (**d**) current density response columnar diagram of the tested analytes compared with DA; (**e**) stability of Au@NiS_2_-FTO for DA detection; (**f**) reproducibility of Au@NiS_2_-FTO biosensors.

Figure 5 shows that the voltage peak value of DA conversion to DA quinone on the Au@NiS_2_-FTO electrode is 0.3 V. Therefore, we use 0.3 V as the working potential; the time current curve of DA on the Au@NiS_2_-FTO electrode was tested, with a testing range from 0.1 μM to 1 mM. The results are shown in Figure 6a. Regression curves of the current at different concentrations and at the same time (100 s) are plotted, as shown in Figure 6a. The linear regression equation is as follows:

I = 0.0000342x + 0.00153 (R^2^ = 0.99), (6)

In the calibration curve, it can be known that the concentration of DA is directly proportional to the absolute value of the oxidation current, and its linear detection range is from 0.1 μM to 1 mM and has a low detection limit (1 nM), indicating high sensitivity of the Au@NiS_2_-FTO electrode for DA detection.

Another key aspect of biosensing technology is the selectivity for common interfering substances. In the extracellular fluid and the serum of the mammalian central nervous system, there are also some reducing agents with oxidation potentials close to that of DA, such as glucose, L-ascorbic acid, uric acid, Ca^2+^, Na^+^, and K^+^, which may have a certain impact on the accurate determination of DA content. It is necessary to distinguish DA from these interfering substances and exploring the anti-interference ability of the Au@NiS_2_-FTO electrode is extremely important [45,46,47,48].

Similarly, we employed a three-electrode system to investigate the interference of glucose, L-ascorbic acid, uric acid, CaCl_2_, NaCl, and KCl on DA. The results are depicted in Figure 6c. Upon adding 0.5 mM DA to the electrolyte, an instantaneous change in the current occurred. Subsequently, the addition of 1 mM glucose, 1 mM L-ascorbic acid, 1 mM uric acid, 1 mM CaCl_2_, 1 mM NaCl, and 1 mM KCl did not result in significant changes in the curve trend. However, upon further addition of 0.5 mM DA, the current exhibited significant fluctuations, indicating that glucose, L-ascorbic acid, uric acid, CaCl_2_, NaCl, and KCl did not induce changes in the current in this environment. To better observe the impact of adding interfering substances on DA, the current change at 0.2 s after adding interfering substances was plotted, as shown in Figure 6d. It is evident in the figure that the current variation in the system after adding interfering substances can be negligible, demonstrating the specificity and sensitivity of the Au@NiS_2_-FTO electrode for DA detection. To test the stability of the Au@NiS_2_-FTO electrode, we conducted tests on five sets of electrodes every 7 days to determine the response signal. The results, as shown in Figure 6e, indicate that the Au@NiS_2_-FTO electrode can maintain over 96% of its activity after 28 days, demonstrating its stability in air. Subsequently, using eight independent electrodes under the same experimental parameters, we measured 1 mM DA, with the results shown in Figure 6f. Calculations show a relative standard deviation (RSD) of 3%, indicating good reproducibility of the biosensor. In conclusion, the proposed Au@NiS_2_-FTO electrode exhibits excellent characteristics, such as anti-interference, stability, and reproducibility, proving its potential for measuring DA in real samples and paving the way for future in vivo testing of Au@NiS_2_-FTO electrodes.

Table 2 summarizes the electrochemical detection performance of various biosensing materials. Compared with other sensing materials, the Au@NiS_2_-FTO prepared in this study demonstrates a much wider detection range and lower limit of detection (LOD).

## 3. Materials and Methods

### 3.1. Material Preparations

NiCl_2_·6H_2_O, CaCl_2_, and NaCl were purchased from Inokai; Na_2_S_2_O_3_·5H_2_O, uric acid (UA), ascorbic acid (AA), the phosphate buffer solution (PBS, pH = 7.3) were purchased from Macklin; and KCl, [K_3_Fe(CN)_6_], [K_4_Fe(CN)_6_] ·3H_2_O, and DA were purchased from Aladdin. All reagents used in the experiments were at an analytical grade level and required no further purification. Deionized water was used throughout the whole experiment.

In the experiment, NiS_2_ was first prepared on the FTO substrate using a traditional hydrothermal method. NiCl_2_·6H_2_O (1.188 g, 0.005 mol) and Na_2_S_2_O_3_·5H_2_O (2.482 g, 0.010 mol) were placed into a 50 mL capacity polytetrafluoroethylene-lined autoclave. Then, 30 mL of ultrapure water was added and stirred for 30 min until dissolved to form a transparent green solution. FTO conductive glass was placed below the liquid level and fixed to the inner wall of the autoclave using heat-resistant tape. The autoclave was maintained at 150 °C for 12 h. Cool to room temperature and take out the sample. The as-prepared NiS_2_ deposited on the FTO was washed repeatedly with distilled water and dried at 65 °C for 12 h.

### 3.2. Preparation of Au@NiS_2_-FTO

A ZHDS400 high-vacuum organic/metal evaporation coating machine (PVD) was utilized for the preparation of Au nanofilm. Place the Au target in the crucible and place NiS_2_-FTO on the sample plate using high-temperature-resistant tape and put it in the evaporation chamber. Heat the sample plate to 80 °C in a vacuum environment and adjust the power to 30 W. Evaporate for 2 min at a rate of 0.42 Å s^−1^, depositing 5 nm of Au on the surface of NiS_2_-FTO. Then, cool the sample to room temperature, deposit 5 nm of Au on the NiS_2_-FTO surface, and obtain Au@NiS_2_-FTO. As a control experiment, 5 nm Au is deposited on the FTO without modification of NiS_2_ using the same method on the coating machine. In this experiment, we chose Au target material with a purity of 99.99%.

### 3.3. Electrochemical Performance Measurement

All the electrochemical tests mentioned in this article were conducted using the CHI660E electrochemical workstation produced by Shanghai Chenhua Instrument Co., Ltd., Shanghai, China. The platinum electrode and Ag/AgCl electrode were produced by Wuhan Gaoshiruilian Technology Co., Ltd., Wuhan, China, and the conductive glass FTO was purchased from Suzhou Shangyang Solar Technology Co., Ltd., Suzhou, China.

Electrochemical tests were conducted using a three-electrode system on a CHI 660E electrochemical workstation. An FTO substrate (10 × 6 mm) coated with NiS_2_ was used as the working electrode (WE), a platinum plate served as the auxiliary electrode (CE), and an Ag/AgCl electrode was used as the reference electrode (RE). Differential pulse voltammetry (DPV) was performed in a 0.1 M PBS (pH = 7.3) solution as the supporting electrolyte.

In the experiment, the Au@NiS_2_-FTO samples prepared by the hydrothermal method and magnetron sputtering deposition were used as working electrodes (WEs). Electrochemical tests, including CV and DPV, were carried out in a 0.1 M PBS (pH = 7.3) solution as the electrolyte, with a scan rate of 100 mV s^−1^ and a potential range of −0.2 to 0.6 V. EIS was carried out in a solution containing 5 mM [K_3_Fe(CN)_6_], 5 mM [K_4_Fe(CN)_6_], and 0.1 M KCl, with a frequency range of 0.01 to 100,000 Hz. Time current curve testing was performed with a voltage input of 0.3 V over a range of 100 s.

## 4. Conclusions

In summary, an Au@NiS_2_-FTO was synthesized on the FTO substrate by a combining hydrothermal and PVD method. The structure and morphology of the samples were characterized by XRD, STEM, TEM, and XPS, which integrally confirmed that Au nanoparticles were uniformly distributed on the surface. The NiS_2_ nanosheets own a large specific surface area and display a significant improvement in electrocatalytic activity towards DA. Moreover, the Au@NiS_2_-FTO electrode showed excellent sensitivity (0.1~1000 μM) and a detection limit of 1 nM for DA, with an electrochemical response time of 0.1 s and outstanding stability. Even after the addition of interfering substances, such as glucose, L-ascorbic acid, uric acid, CaCl_2_, NaCl, and KCl, its potential remained largely unaffected. After 28 days, the Au@NiS_2_-FTO electrode still retains 96% activity, with high reproducibility and an RSD of 3%, demonstrating high resistance to interference, reproducibility, and stability. This work confirms that the Au surface modification is an effective approach to enhancing the sensing ability of DA. This provides direction for DA detection in real serum samples in the future.

## Figures and Tables

**Table 1:** The obtained R_CT_ values correspond to the Faradaic process taking place at various modified electrodes.

Electrode	R_CT_ (Ω)
FTO	153
Au-FTO	83
NiS_2_-FTO	31
Au@NiS_2_-FTO	20

**Table 2:** A comparison of the analytical performance of the proposed sensor with other DA sensors.

Materials	Linear Range (μM)	LOD (nM)	Reference
Au@NiS_2_-FTO	0.1–1000	1	This work
SWCNTs-GCE	0.5–100	190	[49]
ZnO@Au	0.1–500	8.5	[50]
K_2_Fe_4_O_7_/GCE	1–140	22	[51]
Ag/CuO PNBs	0.04–10	7	[52]
Split aptamer sensor	5–50	1000	[53]
pS-BIL MIP PeGE	0.05–250	20	[44]
Gallic acid-RGO/AuNPs	0.01–100.3	2.6	[54]
WO_3_ NPs-GCE	0.1–50, 50–600	24	[55]
CuO	5–40	110	[56]

## Data Availability

Data are contained within the article.

## References

[1] Wang Y., Zhang X., Chen Y., Xu H., Tan Y., Wang S. (2010). Detection of Dopamine Based on Tyrosinase-Fe_3_O_4_ Nanoparticles-chitosan Nanocomposite Biosensor. Am. J. Biomed. Sci..

[2] Luo Q., Su Y., Zhang H. (2022). Sensitive dopamine sensor based on electrodeposited gold nanoparticles and electro-modulated MoS_2_ nanoflakes. J. Iran. Chem. Soc..

[3] Jing W.-J., Li F.-F., Liu Y., Ma R.-N., Zhang W., Shang L., Li X.-J., Xue Q.-W., Wang H.-S., Jia L.-P. (2023). An electrochemical ratiometric biosensor for the detection of dopamine based on an MXene-Au nanocomposite. Chem. Commun..

[4] Yu D., Zhang F., Zhang Y., Lin H., Guo W., Yu K., Qu F. (2023). Heterophase-Structured Cobalt Hydroxide on Partly Reduced Graphene Oxide for Enhanced Dopamine Biosensing. ACS Appl. Eng. Mater..

[5] Gong W., Li J., Chu Z., Yang D., Subhan S., Li J., Huang M., Zhang H., Zhao Z. (2022). A low-cost high-entropy porous CrO/CrN/C biosensor for highly sensitive simultaneous detection of dopamine and uric acid. Microchem. J..

[6] Xu C., Gu C., Xiao Q., Chen J., Yin Z.-Z., Liu H., Fan K., Li L. (2022). A highly selective and sensitive biosensor for dopamine based on a surface molecularly imprinted layer to coordinate nano-interface functionalized acupuncture needle. Chem. Eng. J..

[7] Sun Z., Sun S., Jiang X., Ai Y., Xu W., Xie L., Sun H.-B., Liang Q. (2022). Oligo-layer graphene stabilized fully exposed Fe-sites for ultra-sensitivity electrochemical detection of dopamine. Biosens. Bioelectron..

[8] Yue H.Y., Zhang H.J., Huang S., Lu X.X., Gao X., Song S.S., Wang Z., Wang W.Q., Guan E.H. (2020). Highly sensitive and selective dopamine biosensor using Au nanoparticles-ZnO nanocone arrays/graphene foam electrode. Mater. Sci. Eng. C.

[9] Jiang Y., Wang B., Meng F., Cheng Y., Zhu C. (2015). Microwave-assisted preparation of N-doped carbon dots as a biosensor for electrochemical dopamine detection. J. Colloid Interface Sci..

[10] Liu X., Hou Y., Chen S., Liu J. (2021). Controlling dopamine binding by the new aptamer for a FRET-based biosensor. Biosens. Bioelectron..

[11] Lakard S., Pavel I.-A., Lakard B. (2021). Electrochemical Biosensing of Dopamine Neurotransmitter: A Review. Biosensors.

[12] Akbar F., Kolahdouz M., Larimian S., Radfar B., Radamson H.H. (2015). Graphene synthesis, characterization and its applications in nanophotonics, nanoelectronics, and nanosensing. J. Mater. Sci. Mater. Electron..

[13] Wang K., Liu P., Ye Y., Li J., Zhao W., Huang X. (2014). Fabrication of a novel laccase biosensor based on silica nanoparticles modified with phytic acid for sensitive detection of dopamine. Sens. Actuators B Chem..

[14] Park S.J., Song H.S., Kwon O.S., Chung J.H., Lee S.H., An J.H., Ahn S.R., Lee J.E., Yoon H., Park T.H. (2014). Human dopamine receptor nanovesicles for gate-potential modulators in high-performance field-effect transistor biosensors. Sci. Rep..

[15] Ferlazzo A., Espro C., Iannazzo D., Bonavita A., Neri G. (2023). Yttria-zirconia electrochemical sensor for the detection of tyrosine. Mater. Today Commun..

[16] Ahmed J., Faisal M., Algethami J.S., Alsaiari M., Harraz F.A. (2024). A novel In_2_O_3_-doped ZnO decorated mesoporous carbon nanocomposite as a sensitive and selective dopamine electrochemical sensor. J. Mater. Res. Technol..

[17] Wang S., Ning P., Huang S., Wang W., Fei S., He Q., Zai J., Jiang Y., Hu Z., Qian X. (2019). Multi-functional NiS_2_/FeS_2_/N-doped carbon nanorods derived from metal-organic frameworks with fast reaction kinetics for high performance overall water splitting and lithium-ion batteries. J. Power Sources.

[18] Wang Y., Cai Z., Duan H., Zhang F., Zhai B., Zhao J., Wang X. (2022). Controlled synthesis of rod-like three-dimensional NiS_2_/graphene nanostructures from metal complexes and their application in supercapacitor electrodes. J. Phys. Chem. Solids.

[19] Vellaichamy B., Periakaruppan P., Paulmony T. (2017). Evaluation of a New Biosensor Based on in Situ Synthesized PPy-Ag-PVP Nanohybrid for Selective Detection of Dopamine. J. Phys. Chem. B.

[20] Kajisa T., Li W., Michinobu T., Sakata T. (2018). Well-designed dopamine-imprinted polymer interface for selective and quantitative dopamine detection among catecholamines using a potentiometric biosensor. Biosens. Bioelectron..

[21] Shin J.-W., Yoon J., Shin M., Choi J.-W. (2019). Electrochemical Dopamine Biosensor Composed of Silver Encapsulated MoS_2_ Hybrid Nanoparticle. Biotechnol. Bioprocess Eng..

[22] Dong X., Lu X., Zhang K., Zhang Y. (2012). Chronocoulometric DNA biosensor based on a glassy carbon electrode modified with gold nanoparticles, poly(dopamine) and carbon nanotubes. Microchim. Acta.

[23] Li M., Liu C., Zhao H., An H., Cao H., Zhang Y., Fan Z. (2015). Tuning sulfur doping in graphene for highly sensitive dopamine biosensors. Carbon.

[24] Ghadimi H., Mahmoudian M.R., Basirun W.J. (2015). A sensitive dopamine biosensor based on ultra-thin polypyrrole nanosheets decorated with Pt nanoparticles. RSC Adv..

[25] Arya Nair J.S., Saisree S., Aswathi R., Sandhya K.Y. (2022). Ultra-selective and real-time detection of dopamine using molybdenum disulphide decorated graphene-based electrochemical biosensor. Sens. Actuators B Chem..

[26] Yuan Y., Wang S., Wu P., Yuan T., Wang X. (2022). Lignosulfonate in situ-modified reduced graphene oxide biosensors for the electrochemical detection of dopamine. RSC Adv..

[27] Kang T., Tang T.W., Pan B., Liu H., Zhang K., Luo Z. (2022). Strategies for Controlled Growth of Transition Metal Dichalcogenides by Chemical Vapor Deposition for Integrated Electronics. ACS Mater. Au.

[28] Mondal A., Vomiero A. (2022). 2D Transition Metal Dichalcogenides-Based Electrocatalysts for Hydrogen Evolution Reaction. Adv. Funct. Mater..

[29] Lu T., Wang Y., Cai G., Jia H., Liu X., Zhang C., Meng S., Liu M. (2023). Synthesizability of transition-metal dichalcogenides: A systematic first-principles evaluation. Mater. Futures.

[30] Chen S., Pan Y. (2022). Enhancing catalytic properties of noble metal@MoS_2_/WS_2_ heterojunction for the hydrogen evolution reaction. Appl. Surf. Sci..

[31] Wei C., Cheng C., Cheng Y., Wang Y., Xu Y., Du W., Pang H. (2015). Comparison of NiS_2_ and α-NiS hollow spheres for supercapacitors, non-enzymatic glucose sensors and water treatment. Dalton Trans..

[32] Lu Z., Li Y., Liu T., Wang G., Sun M., Jiang Y., He H., Wang Y., Zou P., Wang X. (2020). A dual-template imprinted polymer electrochemical sensor based on AuNPs and nitrogen-doped graphene oxide quantum dots coated on NiS_2_/biomass carbon for simultaneous determination of dopamine and chlorpromazine. Chem. Eng. J..

[33] Kim J., Byun S., Smith A.J., Yu J., Huang J. (2013). Enhanced Electrocatalytic Properties of Transition-Metal Dichalcogenides Sheets by Spontaneous Gold Nanoparticle Decoration. J. Phys. Chem. Lett..

[34] Atta M.R., Alsulami Q.A., Asnag G.M., Rajeh A. (2021). Enhanced optical, morphological, dielectric, and conductivity properties of gold nanoparticles doped with PVA/CMC blend as an application in organoelectronic devices. J. Mater. Sci. Mater. Electron..

[35] Chen J.-L., Yan X.-P., Meng K., Wang S.-F. (2011). Graphene Oxide Based Photoinduced Charge Transfer Label-Free Near-Infrared Fluorescent Biosensor for Dopamine. Anal. Chem..

[36] Renganathan V., Balaji R., Chen S.-M., Singh V. (2020). The electrochemical determination of hazardous 4-hydroxynitrobenzene using NiS2 decorated graphene oxide nanocomposite in the river water sample. Microchem. J..

[37] Xia N., Deng D., Zhang L., Yuan B., Jing M., Du J., Liu L. (2013). Sandwich-type electrochemical biosensor for glycoproteins detection based on dual-amplification of boronic acid-gold nanoparticles and dopamine-gold nanoparticles. Biosens. Bioelectron..

[38] Silva T.R., Vieira I.C. (2016). A biosensor based on gold nanoparticles stabilized in poly(allylamine hydrochloride) and decorated with laccase for determination of dopamine. Analyst.

[39] Kumar D.R., Baynosa M.L., Dhakal G., Shim J.-J. (2020). Sphere-like Ni_3_S_4_/NiS_2_/MoO_x_ composite modified glassy carbon electrode for the electrocatalytic determination of d-penicillamine. J. Mol. Liq..

[40] Martín M., Salazar P., Villalonga R., Campuzano S., Pingarrón J.M., González-Mora J.L. (2014). Preparation of core–shell Fe_3_O_4_@poly(dopamine) magnetic nanoparticles for biosensor construction. J. Mater. Chem. B.

[41] Baloach Q.-U., Nafady A., Tahira A., Sirajuddin, Sherazi S.T.H., Shaikh T., Arain M., Willander M., Ibupoto Z.H. (2016). An amperometric sensitive dopamine biosensor based on novel copper oxide nanostructures. Microsyst. Technol..

[42] Liu Y., Zhang Y., Wang C., Zeng X., Lei J., Hou J., Huo D., Hou C. (2024). Co Single-Atom Nanozymes for the Simultaneous Electrochemical Detection of Uric Acid and Dopamine in Biofluids. ACS Appl. Nano Mater..

[43] Xie Z., Shao M., Liu Z., Ren X., Gao M., Ma H., Zhang N., Wei Q. (2024). Ultrasensitive aggregation-induced electrochemiluminescence sensor for dopamine detection in polymer hydrogel system. Sens. Actuators B Chem..

[44] Kaya H.K., Cinar S., Altundal G., Bayramlı Y., Unaleroglu C., Kuralay F. (2021). A novel design thia-bilane structure-based molecular imprinted electrochemical sensor for sensitive and selective dopamine determination. Sens. Actuators B Chem..

[45] Li S.-M., Wang Y.-S., Hsiao S.-T., Liao W.-H., Lin C.-W., Yang S.-Y., Tien H.-W., Ma C.-C.M., Hu C.-C. (2015). Fabrication of a silver nanowire-reduced graphene oxide-based electrochemical biosensor and its enhanced sensitivity in the simultaneous determination of ascorbic acid, dopamine, and uric acid. J. Mater. Chem. C.

[46] Rahman S.F., Min K., Park S.-H., Park J.-H., Yoo J.C., Park D.-H. (2016). Highly sensitive and selective dopamine detection by an amperometric biosensor based on tyrosinase/MWNT/GCE. Korean J. Chem. Eng..

[47] Ghosh D., Tabassum R., Sarkar P.P., Rahman M.D.A., Jalal A.H., Islam N., Ashraf A. (2024). Graphene Nanocomposite Ink Coated Laser Transformed Flexible Electrodes for Selective Dopamine Detection and Immunosensing. ACS Appl. Bio Mater..

[48] Karim A., Yasser M., Ahmad A., Natsir H., Wahid Wahab A., Fauziah S., Taba P., Pratama I., Rosalin, Rajab A. (2024). A review: Progress and trend advantage of dopamine electrochemical sensor. J. Electroanal. Chem..

[49] Yang J., Hu Y., Li Y. (2019). Molecularly imprinted polymer-decorated signal on-off ratiometric electrochemical sensor for selective and robust dopamine detection. Biosens. Bioelectron..

[50] Beatto T.G., Gomes W.E., Etchegaray A., Gupta R., Mendes R.K. (2023). Dopamine levels determined in synthetic urine using an electrochemical tyrosinase biosensor based on ZnO@Au core–shell. RSC Adv..

[51] Sun X., Zhang L., Zhang X., Liu X., Jian J., Kong D., Zeng D., Yuan H., Feng S. (2020). Electrochemical dopamine sensor based on superionic conducting potassium ferrite. Biosens. Bioelectron..

[52] Li Y.-Y., Kang P., Wang S.-Q., Liu Z.-G., Li Y.-X., Guo Z. (2021). Ag nanoparticles anchored onto porous CuO nanobelts for the ultrasensitive electrochemical detection of dopamine in human serum. Sens. Actuators B Chem..

[53] Liang Y., Guo T., Zhou L., Offenhäusser A., Mayer D. (2020). Label-Free Split Aptamer Sensor for Femtomolar Detection of Dopamine by Means of Flexible Organic Electrochemical Transistors. Materials.

[54] Tiwari J.N., Vij V., Kemp K.C., Kim K.S. (2016). Engineered Carbon-Nanomaterial-Based Electrochemical Sensors for Biomolecules. ACS Nano.

[55] Anithaa A.C., Lavanya N., Asokan K., Sekar C. (2015). WO_3_ nanoparticles based direct electrochemical dopamine sensor in the presence of ascorbic acid. Electrochim. Acta.

[56] Arvand M., Ghodsi N. (2014). Electrospun TiO_2_ nanofiber/graphite oxide modified electrode for electrochemical detection of l-DOPA in human cerebrospinal fluid. Sens. Actuators B Chem..

